# Intrascrotal Collection in an Acute Pancreatitis: A Case Report and Review of the Literature

**DOI:** 10.1155/2016/7534781

**Published:** 2016-11-01

**Authors:** L. Moens, P. Yengue Yengue, C. Assenmacher

**Affiliations:** ^1^Catholic University of Brussels, Brussels, Belgium; ^2^Department of Gastroenterology, CHwapi, Tournai, Belgium; ^3^Department of Urology, St. Elisabeth Hospital, Brussels, Belgium

## Abstract

*Context*. An inguinoscrotal swelling occurring during an acute pancreatitis is very rare.* Case Report*. We report a case of right inguinoscrotal swelling appearing in connection with an interstitial edematous acute pancreatitis. We have noticed a spontaneous complete reduction of the right inguinoscrotal swelling after 10 days.* Conclusion*. The management of a scrotal swelling should be the least invasive possible method but also the most complete possible method to avoid unnecessary interventions. The exclusion of a pathology that could affect the vital prognosis of the testis remains the absolute priority. An acute scrotum swelling must be carried out by the clinical management by a professional and must be completed with an ultrasonography of the scrotum. Despite all that, if the original etiology of the acute scrotum remains unknown, an abdominopelvic CT scan could provide more details and so could offer a different diagnosis of exclusion, different from the diagnosis of acute idiopathic scrotal edema (AISE). This rare complication of acute pancreatitis reported could be mistaken for a more common pathology. If that complication is identified, it will not require a surgical intervention if there is a correct management of the acute pancreatitis which could justify a broader CT scan.

## 1. Introduction 

Initially the diagnosis of acute pancreatitis is based on the clinical examination associated with the lipase and amylase serum level. Apache II and SIRS are useful. The CT scan is not recommended directly but it is after 72 hours for moderate to severe acute pancreatitis to assess the presence of local complications [1]. There are acute peripancreatic fluids, pseudocysts, necrotic collections, vascular complications, ascitis, and pleural effusion. We have found seventeen cases in the literature (Table 1) presenting local complications extending into the scrotum. In those cases the complication reported had a potential interference for typical urologic differential diagnoses of acute scrotum.

## 2. Case Report

**Figure 1:**
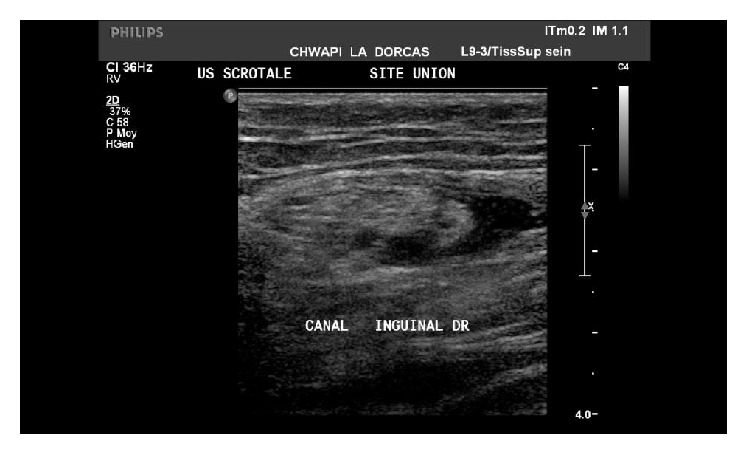
Ultrasound scan demonstrating an infiltration of the inguinal canal fat.

**Figure 2:**
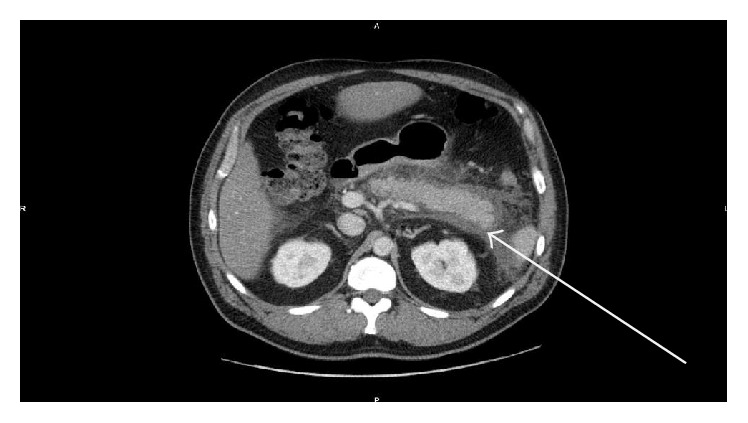
Axial contrast CT scan of the abdomen showing retroperitoneal and peritoneal effusion arising from the tail and the body of the pancreas 4 days after admission.

**Figure 3:**
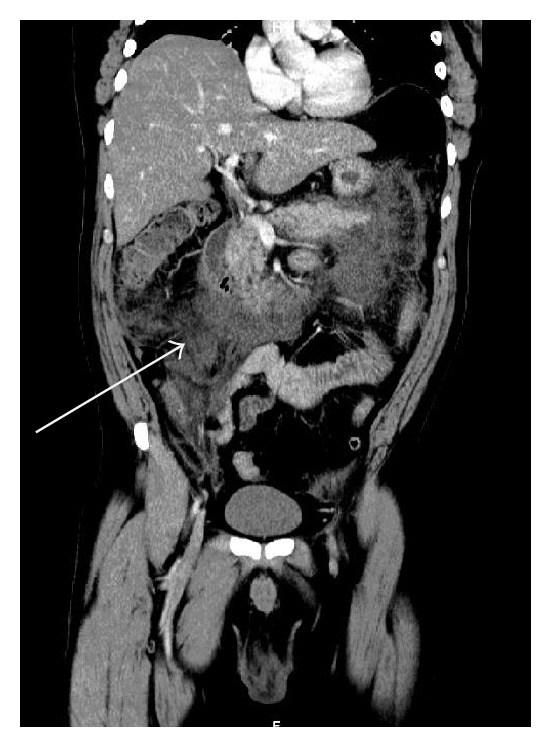
Frontal section contrast CT scan showing retroperitoneal effusion extending from the tail and the body of the pancreas.

**Figure 4:**
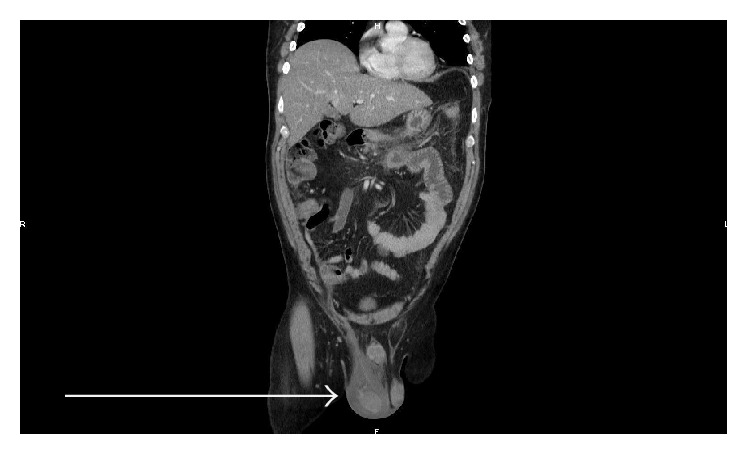
Frontal section contrast CT scan showing involvement of the right scrotum after 4 days of admission.

A 48-year-old male patient with a known diabetic, hypertensive, and hypercholesterolemic status called the Emergency Department for abdominal pain not responding to the paracetamol and butylhyoscine bromide. He complained about increasing broad abdominal pain, nausea, and sweating. His daily treatment was fenofibrate, moxonidine, metformin, and enoxaparin 60 mg. He was a chronic consumer of alcohol. There was an abdominal guarding and pain increased by palpation. His blood test showed high lipase level of 14069 UI/L (regular range: <67 UI/L), a hyperleukocytosis with 14670 units/*μ*L (regular range: 4000 to 10000 units/*μ*L), a hyperneutrophilia with 12264 units/*μ*L (regular range: 2400 to 7500 units/*μ*L), a CRP level of 178 mg/L (normal range: <5.0 mg/L), a YGT serum level of 81 UI/L (normal range for a male: 15–73 UI/L), a triglyceride serum level of 1560 mg/dL, and finally a high blood sugar beyond the patient's regular range. Two days after admission a painful swelling of the right side of his scrotum in an afebrile context with a skin erythema appeared. The ultrasound scan showed an important heterogeneous effusion in the right scrotum and an infiltration of the right inguinal canal fat. The testis was homogenous, well vascularised with a normal size and morphology (Figure 1). The contrast CT scan (Figures 2 and 3) was requested to discover the reason of this infiltration, which showed a peritoneal and retroperitoneal effusion infiltrating the retroperitoneal fat tissues extending from the body and the tail of the pancreas (Figure 2) to the right scrotum (Figure 4), following the iliopsoas muscle and filtering through the right inguinal canal (Figure 3). Finally we diagnosed a right inguinoscrotal swelling related to a mild acute pancreatitis probably induced by an overconsumption of alcohol mixed with a hypertriglyceridemia. He was discharged after 11 days without any specific treatment.

## 3. Discussion 

*“The acute scrotum is a clinical syndrome that is defined as an acute, painful swelling of the scrotum or its contents accompanied by local signs and general symptoms”* [21]. Usually the literatures [21, 22] describe a variety of possible diagnoses as testicular torsion, torsion of testicular appendage, epididymitis, hernia, idiopathic scrotal edema, and tumor (listed in order of relative frequency). In case of acute scrotum it is necessary to exclude the diagnosis of testicular torsion quickly because of a risk of testicular loss in case of delay. The clinical examination seems to be the best way of finding the good diagnosis [21]. If not sufficient, the ultrasound scan with a Doppler is described as the best equipment in case of uncertain diagnosis [20, 22]. As far as our case is concerned, the acute idiopathic scrotal edema (AISE) is often defined as a diagnosis of exclusion because of its unknown etiology. At the present time, AISE is supposed to be a hypersensitivity reaction related to a variant of angioneurotic edema often associated with a hypereosinophilia in 77.8% and a hypervascularization [22].

We thought initially of local causes of unilateral inguinoscrotal swelling. That is the reason why we asked for an ultrasound scan and a Doppler to eliminate the diagnosis of testicle twist, orchiepididymitis, or even inguinal hernia, diagnosis that cannot be postponed. The testis was homogenous, normal sized, and well vascularized. It showed however an infiltration of the right inguinal canal fat with an heterogeneous effusion of the right scrotum. On the basis of the clinical findings and the high serum lipase level, the diagnosis of acute pancreatitis in a context of acute alcohol consumption was made. There were no biological and clinical criteria for a severe acute pancreatitis and so the patient was admitted in gastroenterology. There was no reason for intensive care. It occurred in a context of high consumption of alcohol, a high serum TG level, and a treatment by fenofibrate. We asked for a contrast abdominopelvic CT scan after 72 h which established a direct link between, on the one hand, the retroperitoneal effusion, what comes out the body and the tail of the pancreas and on the other hand the right inguinoscrotal swelling. This fluid dissects the anatomic plans and finds a way from the head and the tail of the pancreas to the scrotum. In this case we supposed that the processus vaginalis is well a remnant.

**Figure 5:**
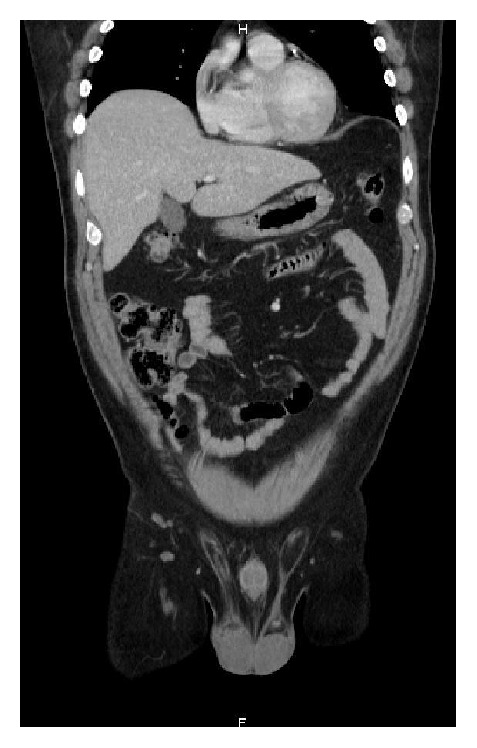
Comparative contrast CT scan after 1 month.

We decided to ensure the patient follow-up with a classic treatment because there was an absence of the pyretic signs usually indicating complications such as necrotizing infected tissues. There was a spontaneous resolution after 10 days (Figure 5).

After having examined the seventeen cases found (Table 1), we can suppose that for acute scrotum complicating an interstitial edematous acute pancreatitis we have the following:

- There is no preference for a side affected.
- The most common etiology for much complicated acute pancreatitis remains the alcohol abuse.
- The etiology was indeed found mainly by the CT scan.
- Concerning the scrotum, the conservative treatment is mostly appropriate.
- The indications reported in the guidelines of acute pancreatitis remain the main source to justify an intra-abdominal invasive treatment (over infected collections not responding to antibiotics, etc.).
- Drainage of intra-abdominal pancreatic collection seems to improve the resolution of acute scrotum.

Because of this rare complication we shall suggest a management combining the pancreatitis and the acute scrotum. By doing so, the number of mistakenly reported pathologies as common may decrease. If the common diagnosis is rejected, the scrotal swelling due to an unknown cause could be identified by mistake as an acute idiopathic scrotal edema (AISE). The low number of case reports describing this complication could explain the fact that there were no appropriate investigations. We suggest confirming the “good diagnosis of AISE” by excluding an intra-abdominal origin with an abdominopelvic CT scan. In this case we have supposed that if we exclude intraabdominal causes, the percentage of hypervascularized and the percentage of hypereosinophilia among “the entity described as a real AISE” could maybe rise.

Yet, we have compared the evolution and the treatment of related cases. The spontaneous resolution seen in our case reinforces the benefits of a noninvasive management whereby we have to remain particularly watchful of possible overcomplications such as infections of necrotized tissues. That is why the patient's supervision in the hospital is still required.

## 4. Conclusion

The management of a scrotal swelling should be the least invasive possible method but also the most complete possible method to avoid unnecessary interventions. The exclusion of a pathology that could affect the vital prognosis of the testis remains the absolute priority. An acute scrotum swelling must be carried out by the clinical management by a professional and must be completed with an ultrasonography of the scrotum. Despite all that, if the original etiology of the acute scrotum remains unknown, an abdominopelvic CT scan could provide more details and so could offer a different diagnosis of exclusion, different from the diagnosis of acute idiopathic scrotal edema (AISE). This rare complication of acute pancreatitis could be mistaken for a more common pathology and if that complication is identified, it will not require a surgical intervention if there is a correct management of the acute pancreatitis which could justify a broader CT scan.

## Figures and Tables

**Table 1:** Literature reports of cases of inguinoscrotal and other extension of pancreatitis local complications.

References + year of publication	Urologic clinical features	Side affected	Etiology of the pancreatitis	Atlanta classification	Invasive scrotal interventions (+intra-abdominal interventions )
[2] 1979	Unknown	Unknown	Unknown	Supposed necrotizing acute pancreatitis	Unknown
[3] 1988	Painful scrotum enlargement	Unknown	Alcohol	Interstitial edematous acute pancreatitis	NO
[4] 1994	Transilluminating hydrocele	Right	Alcohol	Interstitial edematous acute pancreatitis	NO
[5] 1994	Painful and swollen scrotum Inguinal mass	Left	Unknown	Necrotizing acute pancreatitis	NO (+laparotomy)
[6] 1995	Scrotal necrosis	Unknown	Unknown	Necrotizing acute pancreatitis	Excision of the testis and the scrotum
[7] 1996	Scrotal mass, erythematous skin	Unknown	Latrogenic	Necrotizing acute pancreatitis	Invasive scrotal aspiration
[8] 1996	Tender swollen scrotum	Left	Alcohol	Interstitial edematous acute pancreatitis	NO
[9] 2000	Painful inguinal swelling	Right	Alcohol	Interstitial edematous acute pancreatitis	Percutaneous drainage
[10] 2004	Painful scrotal swelling	Right	Alcohol	Interstitial edematous acute pancreatitis	Scrotal fluid punction (+retroperitoneal drainage)
[11] 2006	Inguinoscrotal mass	Left	Alcohol	Interstitial edematous acute pancreatitis	NO
[12] 2006	Scrotal mobile mass	Right	Unknown	Interstitial edematous acute pancreatitis	Percutaneous drainage
[13] 2007	Tender inguinoscrotal swelling	Left	Alcohol	Interstitial edematous acute pancreatitis	(Drainage of the retroperitoneal collection)
[14] 2008	Scrotal exudation, fever	Bilateral	Alcohol	Interstitial edematous acute pancreatitis	Scrotal debridement (+ERCP, pancreatic duct stenting)
[15] 2008	Painful scrotal swelling, fever, funiculitis	Left	Unknown	Interstitial edematous acute pancreatitis	Unknown
[16] 2009	Painful scrotal swelling, macroscopic hematuria	Left	Alcohol	Interstitial edematous acute pancreatitis	NO
[17] 2011	Painful scrotal swelling, discoloration of the scrotum	Left	Alcohol	Necrotizing acute pancreatitis	NO
[18] 2013	Painful tender groin and scrotal swelling	Right	Unknown	Interstitial edematous acute pancreatitis	NO (+drainage paracolic collection)
Our case report	Painful inguinoscrotal swelling + skin erythema	Right	Alcohol, hypertriglyceridemia	Interstitial edematous acute pancreatitis	NO

References + year of publication	Other expressions reported	Side affected	Etiology of the pancreatitis	Atlanta classification	Treatment reported

[19] 1973	Extension to the groin	Right	Alcohol	Interstitial edematous acute pancreatitis	Inguinal canal drainage
[20] 1984	Extension to the groin	Left	Alcohol	Unknown	Laparotomy + peritoneal lavage
[21] 1987	Extension to the thigh and the knee	Left	Gallstones	Interstitial edematous acute pancreatitis	Pseudocyst drainage
[22] 2009	Extension to the psoas muscle	Left	Alcohol	Interstitial edematous acute pancreatitis	Laparotomy + pancreatectomy
